# A predictive tool for the assessment of right ventricular dysfunction in non-high-risk patients with acute pulmonary embolism

**DOI:** 10.1186/s12890-020-01380-8

**Published:** 2021-01-28

**Authors:** Yizhuo Gao, Lianghong Chen, Dong Jia

**Affiliations:** 1 Department of Pulmonary and Critical Care Medicine, Shengjing Hospital of China Medical University, No. 36, Sanhao Street, Shenyang, China; 2 Department of Emergency Medicine, Shengjing Hospital of China Medical University, No. 36, Sanhao Street, Shenyang, China

**Keywords:** Pulmonary embolism, Computerized tomography, Pulmonary hypertension, N-terminal pro-brain natriuretic peptide, Cardiac troponin I

## Abstract

**Background:**

Rapid and accurate identification of right ventricular (RV) dysfunction is essential for decreasing mortality associated with acute pulmonary embolism (PE), particularly for non-high-risk patients without hypotension on admission. This study aimed to develop a rapid and accurate tool for predicting the risk of RV dysfunction in non-high-risk patients with acute PE.

**Methods:**

The medical records of non-high-risk patients with acute PE admitted to Shengjing Hospital of China Medical University between January 2011 and May 2020 were retrospectively analysed. The primary outcome of this study was RV dysfunction within 24 h after admission. The enrolled patients were randomized into training or validation sets as a ratio of 2:1. In the training set, a nomogram was developed, and the consistency was corroborated in the validation set. The areas under the receiver operating characteristic curves (AUCs) and 95% confidence intervals (CIs) were calculated.

**Results:**

A total of 845 patients were enrolled, including 420 men and 425 women with an average age of 60.05 ± 15.43 years. Right ventricular dysfunction was identified in 240 patients (28.40%). The nomogram for RV dysfunction included N-terminal pro-brain natriuretic peptide, cardiac troponin I, and ventricular diameter ratios, which provided AUC values of 0.881 in the training dataset (95% confidence interval (CI): 0.868–0.898, *p* < 0.001) and 0.839 in the validation set (95% CI: 0.780–0.897, *p* < 0.001). The predictive tool was published as a web-based calculato (https://gaoyzcmu.shinyapps.io/APERVD/).

**Conclusions:**

The combination of CT and laboratory parameters forms a predictive tool that may facilitate the identification of RV dysfunction in non-high-risk patients with acute PE.

## Background

Patients suffering from acute pulmonary embolism (PE) have a wide range of short-term prognoses [1]. High-risk patients with acute PE display overt symptoms of shock and may deteriorate rapidly. However, the majority of patients with acute PE are non-high-risk and do not present with shock [2]. However, some non-high-risk patients with right ventricular (RV) dysfunction also experience adverse in-hospital outcomes, despite the appearance of being low-risk on admission [3, 4]. Therefore, the detection of RV dysfunction is essential to improve the clinical management and decrease mortality in non-high-risk patients with acute PE [5, 6].

In acute PE, the standard diagnostic method for RV dysfunction is echocardiography; however, an experienced sonographer is not always available [1]. The assessment of RV function guides clinical decisions and the prioritization of patient monitoring to prevent adverse in-hospital outcomes of at-risk patients [5]. Clinicians are required to make prompt clinical decisions due to the rapid disease progression of patients with acute PE after admission [7]. Therefore, developing a fast and accurate tool to predict the risk of RV dysfunction in non-high-risk patients is beneficial for risk stratification.

Although some methods for predicting RV dysfunction have been applied in the clinical setting using laboratory and computed tomography (CT) parameters, their utilization and predictive values remain controversial [1, 8–10]. Incorporating clinical, laboratory, and CT parameters to build a predictive tool for RV dysfunction may advance the discriminatory power, although the method of this combination still needs to be optimized. In this study, the clinical, laboratory, and CT data were collected from the medical records of non-high-risk patients with acute PE and used to develop a predictive tool for RV dysfunction that is simple and user-friendly. A nomogram was built and developed into a web-based calculator to predict the risk of RV dysfunction.

## Methods

### Study population

This was a retrospective study in Shengjing Hospital of China Medical University from January 2011 to May 2020.The non-high-risk acute PE patients who were diagnosed by CT pulmonary angiography (CTPA) and more than 18 years were selected preliminarily on admission into our hospital. The definition of non-high-risk acute PE patients was referred to 2019 European Society of Cardiology guidelines for acute PE [2]. Patients who were pregnant, received thrombolysis treatment before admission, or had missing data including CTPA, cardiac troponin I (c-Tn I), N-terminal pro-brain natriuretic peptide (NT-pro BNP) or echocardiography, were excluded from this study.

### Clinical and laboratory parameters

The demographic characteristics and baseline data were collected. The systolic pressure, heart rate and arterial oxyhemoglobin saturation were obtained on admission records together with a detailed medical history. Serum c-Tn I and NT-pro BNP were measured within 2 h followed by a diagnostic CTPA in patients or by diagnosis of the diseases when patients with severe symptoms including dyspnoea, chest pain, haemoptysis, and other related symptoms on admission. The c-Tn I and NT-pro BNP data were collected.

### Computed tomography acquisition

A 64-deector-row CT was used to perform the pulmonary arteriography the details of the equipment was listed as follow: KV-120; Toshiba Medical Systems Corporation, Tokyo, Japan; The acquisition parameter was listed as 1.0 mm sections with 370 mA and 120 kV. Approximate 100 mL contrast of iodized non-ionic type was injected into elbow vein by an automatic dual-tube high-pressure injector from the thoracic inlet to the upper abdomen at a rate of 4 ml/s.

### Reconstruction of computed tomography parameters

In consideration of suitability for clinical practice, the items of CTPA parameters were reconstructed. Clot location was defined as three types: Saddle-main pulmonary artery (MPA) embolism, MPA embolism and non-MPA embolism. MPA was extracted from CTPA data firstly from the inlet of pulmonary artery trunk to the outlet of right and left side MPA [11]. If thrombus located at the bifurcation section and extension into two side right and left MPA, Saddle-MPA embolism was identified; If thrombus located in MPA section and no thrombus located at bifurcation section of MPA, MPA embolism was identified. If no thrombus located in MPA, non-MPA embolism was identified [12, 13] (Additional file 1).Two planes were reconstructed for measurement cardiac size including short-axis plane and 4 chamber view. At short-axis plane, the RV and left ventricle (LV) maximal diameters were measured between the free wall of the ventricle and interventricular septum, perpendicular to interventricular septum respectively [8].The ratio of RV-to-LV at short-axis plane was calculated (Additional file 2). At the 4 chamber view, measurement RV, LV, right atrium (RA) and left atrium (LA) maximal diameters were perpendicular to interventricular and interatrial septum respectively [14].The relative ratios of the RV-to-LV and RA-to-LA were calculated at 4 chamber view (Additional file). All the parameters were reconstructed and measured using a Mimics Medical software (version 19.0, Mimics Medical software, Leuven, Belgium).

### Primary endpoint

The primary endpoint of this study was defined as occurring RV dysfunction within 24 h of admission by an echocardiography by an IE Elite ultrasound machine (Philips) equipped with an transducer (frequency conversion 1–5 MHz). Occurrence of anyone or more appearances was defined as RV dysfunction: RV diameter > 30 mm from 4-chamber or parasternal view. At end-systole. RV/LV 4-chamber diameter ratio > 0.9 RV hypokinesia occurred at free wall. The jet of tricuspid regurgitation’s velocity increased at apical 4-chamber view [15, 16]. All echocardiographic procedures were performed by ultrasound specialists as the same standard.

### Development of a predictive tool for right ventricular dysfunction

The enrolled patients were randomized into a training set and a validation set as a ratio of 2:1.The training set was used to develop a nomogram for predicting RV dysfunction as the standard of Transparent Reporting of a multivariable prediction model for Individual Prognosis or Diagnosis (TRIPOD) standard [17]. The biochemical and clinical data, and CTPA parameters in the training set were analysed together. To avoid collinearity and to screen the parameters [18], a classification and regression tree (CART) analysis was used for converting the continuous variables into dichotomous variables with optimal cut-off values according to the endpoint. A logistic regression model was constructed with the variables screened by CART. A nomogram was developed from the logistic regression model of the training set. The discriminatory power for RV dysfunction was evaluated and the consistency of the nomogram was assessed with the validation set. This predictive tool was applied as a web-based calculator based on the nomogram.

### Statistical analysis

In this study, continuous data is expressed as mean ± standard deviation and tested with a Student’s t test. The categorical data is expressed as number (%) and compared with a Chi-square test. CART analysis was used to dichotomize each parameter and identify significant parameter [19, 20].

To evaluate the correlation to RV dysfunction, univariate and multivariate logistic regression analysis were performed. The odds ratios (ORs) and 95% confidence intervals (CIs) were calculated from the logistic regression analysis. The nomogram for predicting RV dysfunction was created by regression coefficient of each variable based on the multivariate logistic regression above. The variable with the highest weight was defined as the range from 0 to 100 points. The other variable scores was calculated based on this variable with the highest weight. Each variable ranged from 0 to 100 points. The scores of each variable were added into total scores. [21]. By the total scores from the nomogram, the risk groups were divided further by another CART analysis. The predictive ability of the nomogram was estimated by the concordance index (C-index) and calibration curve with bootstrap by resampling 1000 times [17, 21, 22]. To evaluate the sensitivity, specificity, positive predictive value (PPV), negative predictive value (NPV), a receiver operating characteristic (ROC) curve was used and the area under the ROC curve (AUC) was calculated. The calibration curve was used to assess the consistency between actual incidence and predicted incidence of the nomogram [22]. A decision curve analysis (DCA) was performed to evaluate the applicability [21]. Statistical significance was set at *p* < 0.05 and all analyses were performed using R software (version 4.0.1; R Foundation https://www.r-project.org).

## Results

### Demographics and baseline characteristics

The data of 902 consecutive non-high-risk patients were considered for this study. After further screening, 57 patients were excluded (3 pregnant patients, 5 patients treated with thrombolysis before admission to the hospital, and 49 patients missing the transthoracic echocardiography, NT-pro BNP, or c-Tn I data). In addition, 53 patients were identified as having a pre-existing heart disease, including 24 patients with definitive heart failure, 21 patients with cor pulmonale, and 8 patients with coronary artery disease. RV dysfunction was diagnosed in 240 patients (110 males and 130 females, average age: 62.50 ± 14.28 years) according to the echocardiography results. A total of 605 patients (310 males and 295 females, average age: 59.07 ± 15.76 years) were not found to have RV dysfunction. The patients with RV dysfunction had significantly higher heart rate, c-Tn I, and NT-pro BNP (all *p* < 0.001) than patients without RV dysfunction. Patients with RV dysfunction were also more likely to have MPA embolisms and saddle-MPA embolisms and less likely to have non-MPA embolisms than patients without RV dysfunction (all *p* < 0.001). Furthermore, patients with RV dysfunction had a significantly greater mean RV short-axis diameter, RV 4-chamber diameter, RA 4-chamber diameter, RV/LV short-axis diameter ratio, RV/LV 4-chamber diameter ratio, and RA/LA 4-chamber diameter ratio (all *p* < 0.001) than patients without RV dysfunction. However, patients with RV dysfunction had significantly lower mean values of LV short-axis diameter, LV 4-chamber diameter, and LA 4-chamber diameter than patients without RV dysfunction (all *p* < 0.001) (Table 1).

**Table 1:** Patient baseline characteristics

	Total (n = 845)	RV dysfunction (n = 240)	No RV dysfunction (n = 605)	*p* value
Sex (male)	420 (49.7%)	110 (45.8%)	310 (51.2%)	0.16
Age (years)	60.05 ± 15.43	62.50 ± 14.28	59.07 ± 15.76	0.002
Heart rate (beats/min)	86.57 ± 17.81	95.99 ± 20.19	82.84 ± 15.25	< 0.001
Systolic pressure(mmHg)	124.33 ± 18.26	122.58 ± 21.38	125.03 ± 16.83	0.073
c-Tn I (μg/L)	0.11 ± 0.47	0.19 ± 0.42	0.073 ± 0.48	< 0.001
NT-pro BNP (pg/mL)	1547.12 ± 3652.06	2858.93 ± 4607.22	1024.73 ± 3046.80	< 0.001
MPA embolism	209 (24.7%)	119 (49.6%)	90 (14.9%)	< 0.001
non-MPA embolism	636 (75.3%)	121 (50.4%)	515 (85.1%)	< 0.001
Saddle-MPA embolism	62 (7.3%)	40 (16.7%)	22 (3.6%)	< 0.001
RV short-axis diameter (mm)	39.21 ± 7.42	43.97 ± 8.13	37.32 ± 6.40	< 0.001
LV short-axis diameter (mm)	40.76 ± 7.30	36.42 ± 7.66	42.48 ± 6.40	< 0.001
RV 4-chamber diameter (mm)	36.38 ± 7.37	40.08 ± 8.10	34.91 ± 6.51	< 0.001
LV 4-chamber diameter (mm)	39.47 ± 7.36	35.95 ± 7.24	40.87 ± 6.93	< 0.001
RA 4-chamber diameter (mm)	45.44 ± 9.34	50.12 ± 9.49	43.58 ± 8.61	< 0.001
LA 4-chamber diameter (mm)	34.83 ± 8.52	32.81 ± 8.40	35.64 ± 8.44	< 0.001
RV/LV short-axis diameter ratio	1.00 ± 0.30	1.26 ± 0.37	0.89 ± 0.18	< 0.001
RV/LV 4-chamber diameter ratio	0.96 ± 0.31	1.17 ± 0.42	0.87 ± 0.19	< 0.001
RA/LA 4-chamber diameter ratio	1.39 ± 0.52	1.65 ± 0.63	1.29 ± 0.40	< 0.001

c Tn-I, cardiac troponin I; NT-pro BNP, N-terminal pro-brain natriuretic peptide; MPA, main pulmonary artery; RV, right ventricle; LV, left ventricle; RA, right atrium; LA, left atrium

### Variable selection

**Fig. 1:**
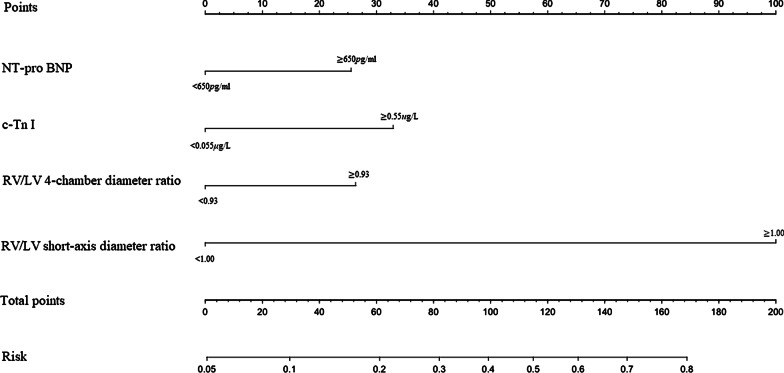
The nomogram for predicting the risk of RV dysfunction. c Tn-I, cardiac troponin I; NT-pro BNP, N-terminal pro-brain natriuretic peptide; RV, right ventricle; LV, left ventricle

Four variables were considered significant predictors of RV dysfunction and were dichotomized: NT-pro BNP (≥ 650 pg/mL vs. < 650 pg/mL), c-Tn I (≥ 0.055 μg/L vs. < 0.055 μg/L), RV/LV short-axis diameter ratio (≥ 1.00 vs. < 1.00), and RV/LV 4-chamber diameter ratio (≥ 0.93 vs. < 0.93). A multivariate logistic regression analysis using the training dataset revealed that NT-pro BNP (OR: 1.93, 95% CI: 1.13–3.31, *p* < 0.001), c-Tn I (OR: 2.33, 95% CI: 1.28–4.25, *p* < 0.001), RV/LV short-axis diameter ratio (OR: 13.12, 95% CI: 7.71–22.34), and RV/LV 4-chamber diameter ratio (OR: 1.97, 95% CI: 1.15–3.38, *p* < 0.001) each independently predicted RV dysfunction (Table 2). These variables were used to develop a nomogram (Fig. 1).

**Table 2:** Parameters used in the nomogram to predict RV dysfunction in the training set

	Univariate analysis		Multivariate analysis	
	OR (95% CI)	*p* value	OR (95% CI)	*p* value
NT-pro BNP (≥ 650 vs. < 650 pg/mL)	5.00 (3.38–7.41)	< 0.001	1.93 (1.13–3.31)	0.017
c-Tn I (≥ 0.055 vs. < 0.055 μg/L)	5.96 (3.87–9.19)	< 0.001	2.33 (1.28–4.25)	0.0056
RV/LV short-axis diameter ratio (≥ 1.00 vs. < 1.00)	23.76 (14.73–38.33)	< 0.001	13.12 (7.71–22.34)	< 0.001
RV/LV 4-chamber diameter ratio (≥ 0.93 vs. < 0.93)	6.63 (4.34–10.11)	< 0.001	1.97 (1.15–3.38)	0.014

NT-pro BNP, N-terminal pro-brain natriuretic peptide; c-Tn I, cardiac troponin I; RV, right ventricle; LV, left ventricle; OR, odds ratio; CI, confidence intervals

### Performance of the nomogram in the training and validation set

**Fig. 2:**
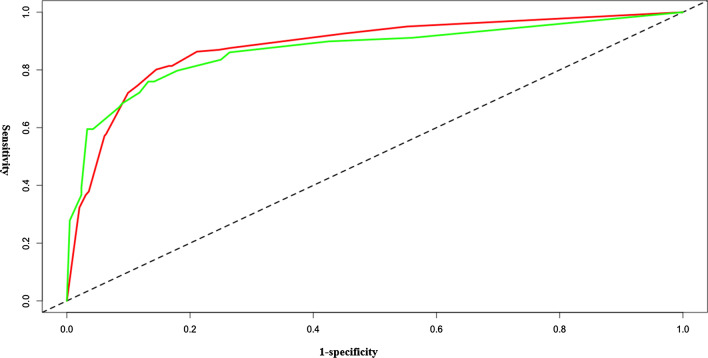
The ROC curves for predicting RV dysfunction. The AUC value for predicting right ventricle dysfunction is 0.881 in the training set (95% CI: 0.868–0.898, *p* < 0.001) and 0.839 in the validation set (95% CI: 0.780–0.897, *p* < 0.001). The training set is represented by the red line and the validation set is represented by the green line. RV, right ventricular; ROC, receiver operating characteristic; AUC, area under the receiver operating characteristic

**Fig. 3:**
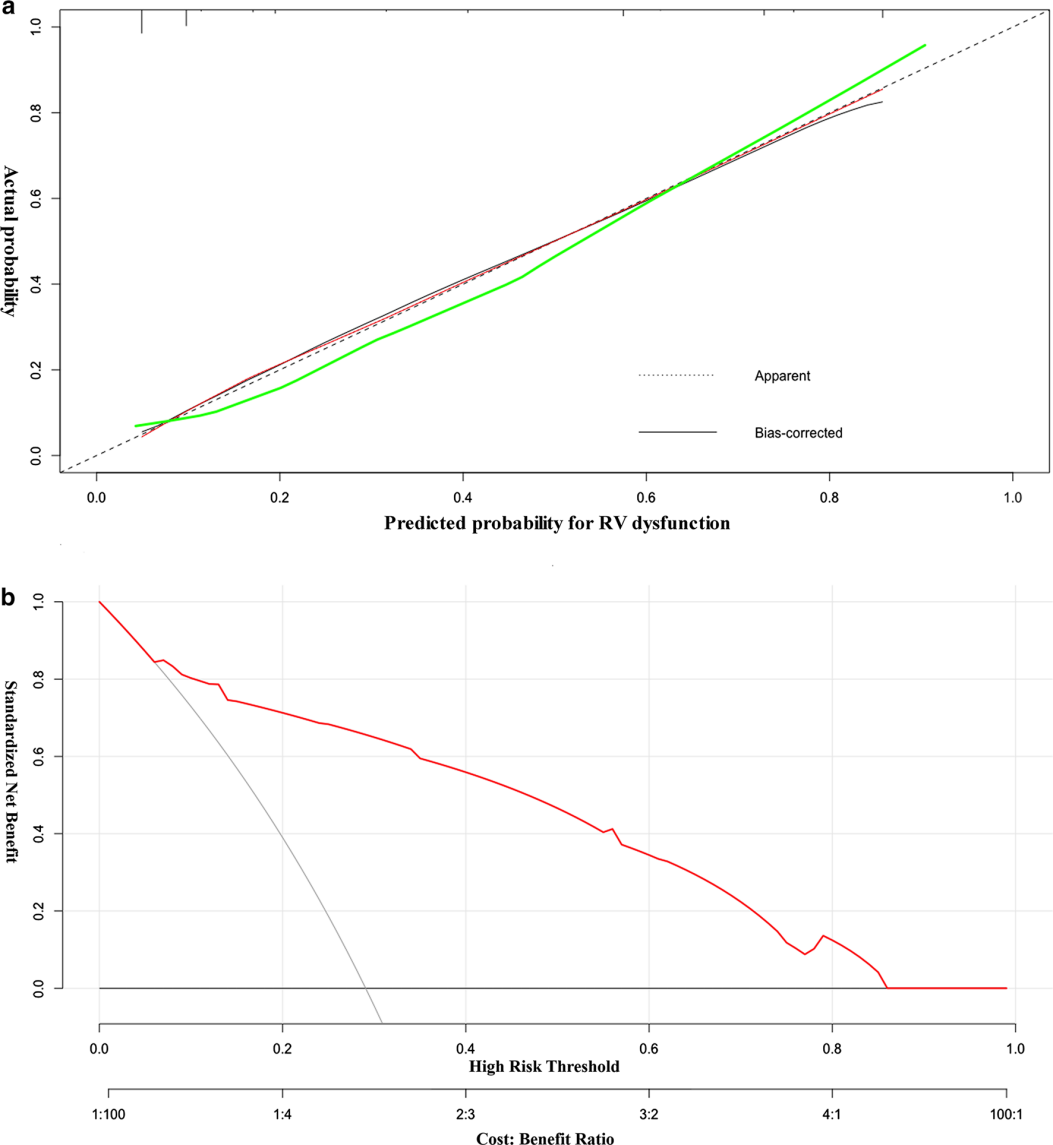
Calibration curve and decision curve for the nomogram. **a** Calibration curve for the nomogram predicting RV dysfunction. The training dataset is represented by the red line and the validation set is represented by the green line. **b** Decision curve analysis for the nomogram comparing the net benefit for predicting RV dysfunction (net benefit: 0.07–0.85) RV, right ventricular

The four variables in the nomogram resulted in favourable C-index values in the training set (C-index: 0.881, 95% CI: 0.868–0.898) and in the validation set (C-index: 0.839, 95% CI: 0.780–0.897) (Fig. 2). The calibration curve also revealed good agreement between the nomogram’s prediction and the actual outcomes (Fig. 3a). A decision curve analysis from the nomogram comparing the net benefit for predicting RV dysfunction ranged from 0.07 to 0.85 (Fig. 3b). The PPV and NPV of the nomogram were 69.35% and 91.30%, respectively.

### Development of individual risk assessment for right ventricular dysfunction

The nomogram for predicting RV dysfunction was used to develop a web-based calculator (https://gaoyzcmu.shinyapps.io/APERVD/) that assigned patients to a high-risk (≥ 92 points) or low-risk group (< 92 points) (Additional file 3). The QR codes in the lower right corner of the calculator webpage can be used to extract the results to mobile electronic equipment.

## Discussion

A predictive tool for RV dysfunction was developed using NT-pro BNP, c-Tn I, and ventricular ratios in the 4-chamber view and short-axis plane. Clinicians can use this tool to predict the risk of RV dysfunction in non-high-risk patients with acute PE. Previous studies assessed the predictive value of a combination of laboratory and CT parameters to predict RV dysfunction; however, these studies had some limitations [10, 23]. We developed a novel method to predict RV dysfunction based on clinical, laboratory and imaging parameters. In comparison with previous studies, this study redefined the threshold of c-Tn I and NT-pro BNP, and incorporated two ventricular ratios with CT parameters to develop a predictive tool using CART analysis. Refining and screening predictors were pivotal in improving the predictive value of our study [18]. This study selected predictors using a CART analysis, which is a purposeful cluster analysis, controlling for confounders and heterogeneity [19, 24]. Through this analysis, four variables were found to be independent significant predictors of RV dysfunction, and these four predictors were developed into a nomogram. The CART analysis was used to develop optimal cut-off values for c-Tn I, NT-pro BNP, RV/LV 4-chamber size, and RV/LV short-axis diameter ratio. This statistical method contributed to the superior predictive value for RV dysfunction.

Increased afterload and contractility of the right ventricle are the main reasons for acute RV dysfunction [25]. These pathophysiological mechanisms, both involved in acute PE and RV dysfunction, worsen LV function through the compression of the interventricular septum, resulting in decreased blood return to the LV [23]. Right ventricular dysfunction is related to increased pressure in the pulmonary circulation, ranging from mild to severe pulmonary hypertension (PH). Mild PH leads to RV dilation without compressing the interventricular septum, whereas severe PH causes RV dilation with compression of the interventricular septum [26]. Regardless of the severity of PH, RV dilation is a critical component of PH pathophysiology. In contrast, patients with PE without RV dysfunction are able to compensate for the increased pulmonary pressure caused by PE [27]. This compensation does not result in a decrease of blood return to the LV [2], therefore, PH does not progress [26]. This may be the main culprit of the clinical deterioration observed in non-high-risk patients with acute PE and RV dysfunction, rendering the identification of RV dysfunction essential to improve the therapeutic approach of these patients [3, 5].

The short-axis plane in CT is widely used in the evaluation of RV dilation [8], and its RV/LV diameter ratio is the accepted predictor for RV dysfunction [2, 9]. In this study, RV/LV short-axis diameter ratio was the principle factor in the nomogram. The 4-chamber view was reconstructed perpendicularly to the ventricular septum. This reconstructed CT plane reflects the interaction between the RV, interventricular septum, and LV. In PH, the compression from the right to the left ventricle is transmitted by the interventricular septum. In this study, the increase in the RV/LV ratio was due to this compression. In summary, the RV/LV short-axis diameter and RV/LV 4-chamber diameter ratios were included in our predictive tool for RV dysfunction as a response of RV dilation and RV to LV compression, respectively. Cardiac volume may also predict RV dysfunction; however, we aimed to develop a predictive tool with application for clinicians, therefore, we did not incorporate cardiac volume parameters.

Other clinical parameters, including increased heart rate and decreased systolic pressure, are predictors of poor short-term prognosis in patients with acute PE [2]; however, they were not included in our predictive tool. Although the occurrence of RV dysfunction leads to the decompensation of pulmonary circulation [25], compensation of the systemic circulation maintains cardiac output. A presentation of tachycardia and hypotension represent severe RV dysfunction in patients with acute PE; however these symptoms are absent in patients with mild RV dysfunction. Therefore, heart rate and systolic pressure were not used as predictors in our predictive tool. Elevated NT-pro BNP and c-Tn I are caused by increased RV tension [9, 23]. It has been reported that NT-pro BNP, c-Tn I, and CT parameters improve the accuracy of the diagnosis of RV dysfunction in comparison with a single test [9]; however, no accepted method that combines these parameters exists. This is a study to develop an accurate nomogram that combines these parameters, and clinicians can use this tool to predict the risk of RV dysfunction in patients with acute PE at admission.

## Limitations

This study is not without limitations. Firstly, the retrospective design of this study limited the statistical strength. To overcome this limitation, we enrolled a large number of patients with acute PE with RV dysfunction, as well as patients without RV dysfunction as a control group. Secondly, we developed a predictive tool using randomized grouping; however, the clinical validity of this predictive tool requires further verification. Thirdly, while transthoracic echocardiography with uniform criteria was conducted to diagnose RV dysfunction, all subjective bias may not have been eliminated.

## Conclusion

A predictive tool for RV dysfunction in non-high-risk patients with acute PE using the ventricular ratio on the short-axis plane and 4-chamber view, c-Tn I, and NT-pro BNP is presented in this study. Using this tool, clinicians can identify patients with RV dysfunction rapidly and accurately upon admission to the hospital.

## Supplementary information

**Additional file 1.** Clot location (CTPA images describing the classifications of the clot locations).

**Additional file 2.** Measurement of cardiac diameter (CTPA images describing the methods used to measure the heart dimensions used in this study).

**Additional file 3.** Web-based calculator for predicting RV dysfunction (an image of the web-based calculator developed from the nomogram).

## Data Availability

The data and material during the current study are available from the corresponding author on reasonable request.

## Abbreviations

PE: Pulmonary embolism
CT: Computed tomography
c-Tn I: Cardiac troponin I
NT-pro BNP: N-terminal pro-brain natriuretic peptide
RV: Right ventricular
LV: Left ventricular
RA: Right atrium
LA: Left atrium
MPA: Main pulmonary artery
CART: Classification and regression tree
ORs: Odds ratios
C-index: Concordance index
ROC: Receiver operating characteristic
PPV: Positive predictive value
NPV: Negative predictive value
AUC: Area under the receiver operating characteristic curve
DCA: Decision curve analysis
PH: Pulmonary hypertension
