# Supplementary material for: A predictive tool for the assessment of right ventricular dysfunction in non-high-risk patients with acute pulmonary embolism
Source: BMC Pulm Med. 2021 Jan 28;21:42. doi: 10.1186/s12890-020-01380-8 (PMC7842037; doi:10.1186/s12890-020-01380-8)
Supplement: Supplementary file 1 — Additional file 1. Clot location (CTPA images describing the classifications of the clot locations). [file 12890_2020_1380_MOESM1_ESM.docx]

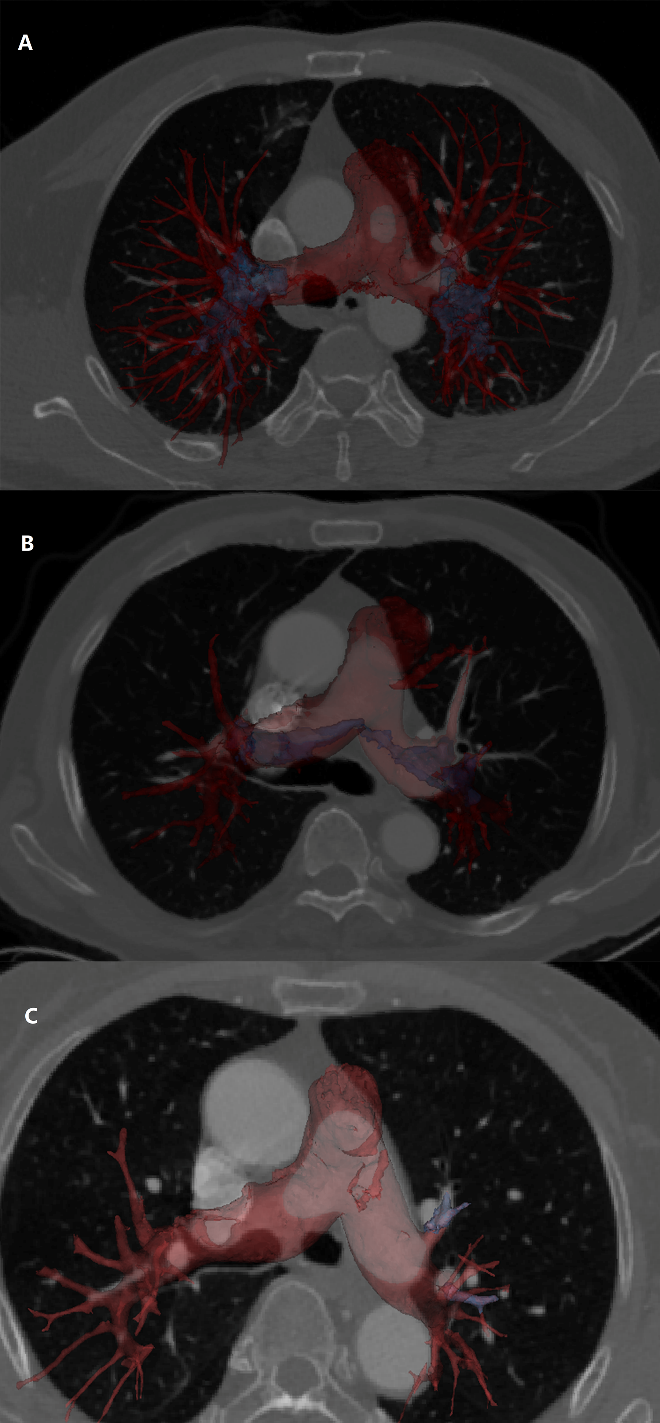


**Additional file 1:** Clot location. The reconstruction in red indicates MPA and the reconstruction in blue indicates the clot. Based on the relative location between the MPA and clot, the clot location is divided into MPA embolism (clot in MPA), saddle-MPA embolism (clot at bifurcation of MPA) and non-MPA embolism (clot at peripheral pulmonary artery).

1. **MPA embolism;**
2. **Saddle-MPA embolism;**
3. **non-MPA embolism.**

MPA, main pulmonary embolism
