# Supplementary material for: A predictive tool for the assessment of right ventricular dysfunction in non-high-risk patients with acute pulmonary embolism
Source: BMC Pulm Med. 2021 Jan 28;21:42. doi: 10.1186/s12890-020-01380-8 (PMC7842037; doi:10.1186/s12890-020-01380-8)
Supplement: Supplementary file 2 — Additional file 2. Measurement of cardiac diameter (CTPA images describing the methods used to measure the heart dimensions used in this study). [file 12890_2020_1380_MOESM2_ESM.docx]

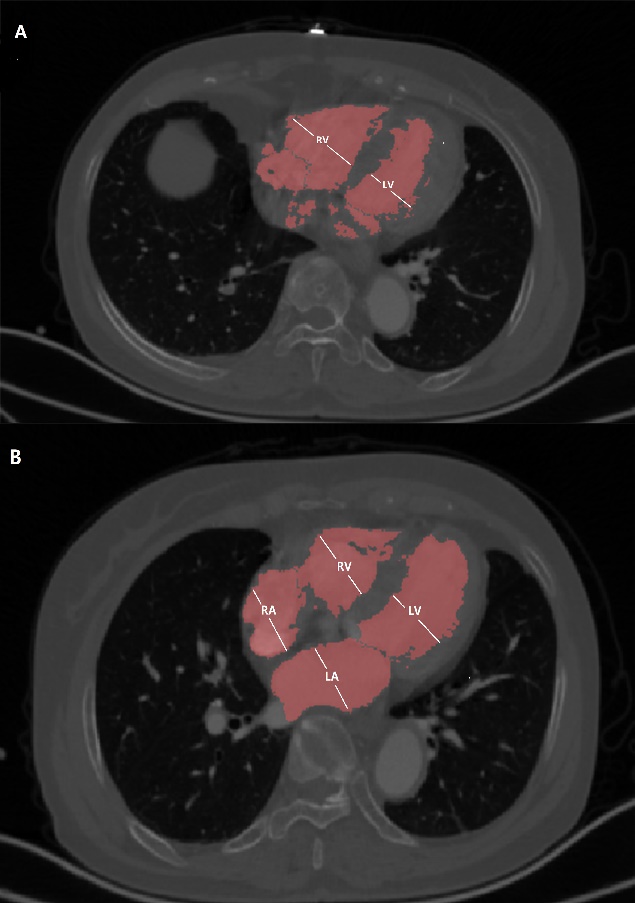


**Additional file 2:** Measurement of cardiac diameter. In short-axis, measurement diameter of RV and LV are illustrated and at 4-chamber view the measurement diameter of RV, LV, RA and LA are illustrated.

B. RV and LV diameter at short-axis diameter;

1. RV, LV, RA and LA diameter at 4-chamber view.

RV, right ventricle; LV, left ventricle; RA, right atria; LA, left atria.
