# Supplementary material for: A predictive tool for the assessment of right ventricular dysfunction in non-high-risk patients with acute pulmonary embolism
Source: BMC Pulm Med. 2021 Jan 28;21:42. doi: 10.1186/s12890-020-01380-8 (PMC7842037; doi:10.1186/s12890-020-01380-8)
Supplement: Supplementary file 3 — Additional file 3. Web-based calculator for predicting RV dysfunction (an image of the web-based calculator developed from the nomogram). [file 12890_2020_1380_MOESM3_ESM.docx]

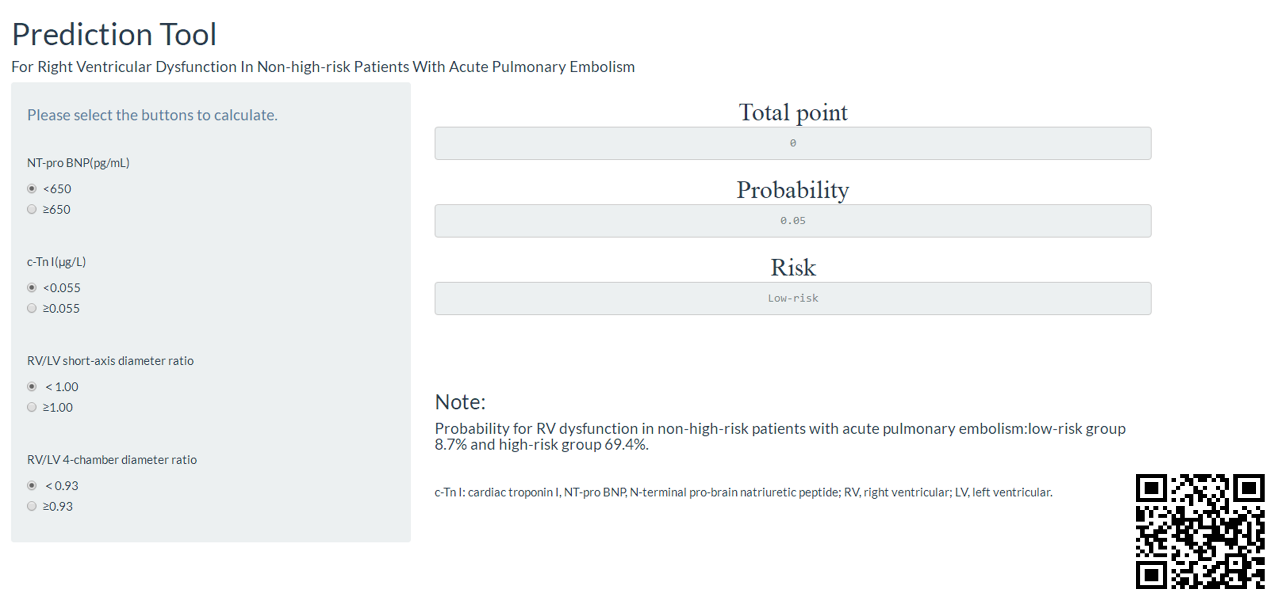


**Additional file 3:** The web-based calculator for predicting RV dysfunction. By access

<https://gaoyzcmu.shinyapps.io/APERVD/> or scanning QR code at lower right corner, the risk of RV dysfunction can be calculated by entering the items in the right column.

c Tn-I: cardiac troponin I; NT-pro BNP, N-terminal pro-brain natriuretic peptide; RV, right ventricle; LV, left ventricle
